# Effect of Using a Plastic Stent with Apically Repositioned Flap in Peri-Implant Soft Tissue Augmentation: A Randomized Controlled Clinical Trial

**DOI:** 10.1155/2021/5590400

**Published:** 2021-05-18

**Authors:** Ahmed Hamdy, Hala K. Abd El Gaber, Dalia M. Ghalwash, Waleed M. Abbas

**Affiliations:** ^1^Oral Medicine and Periodontology, Faculty of Dentistry, The British University in Egypt (BUE), Cairo, Egypt; ^2^Oral Medicine and Periodontology, Faculty of Dentistry, Ain Shams University, Cairo, Egypt

## Abstract

**Objectives:**

This study aimed to clinically assess and compare the width of peri-implant keratinized mucosa following the use of a readymade plastic stent with apically repositioned flap versus conventional apically repositioned flap with papillary sparing incisions during single-stage implant placement protocol.

**Materials and Methods:**

A total of 20 patients were enrolled in this study. In the test group, a prefabricated implant-retained stent was clipped on the healing abutment after implant surgery to reposition the keratinized tissue buccoapically. In the control group, simple interrupted sutures were applied instead of using a stent. After the surgical procedure, the width of the buccal keratinized mucosa was measured at the mesial, middle, and distal aspects of the healing abutment. The change in the width of the buccal keratinized mucosa was assessed at 3 months and 6 months.

**Results:**

No statistically significant difference was found between the stent group and control group in 6 months' interval where *p*=0.840, where both groups showed the same mean value of 4.70 ± 0.35 and 4.70 ± 0.63, respectively. The percent of change in the width of KM was found to be higher in the stent group than in the control group with no statistical significance.

**Conclusion:**

The use of a readymade plastic stent in combined full/partial-thickness apically repositioned flap shows to be effective in increasing the width of KM compared to the conventional technique. This trial is registered with NCT03754894.

## 1. Introduction

The soft tissue barrier at the transmucosal passage of the implant unit plays an important role in the maintenance of stability and function of the load-bearing implant. The presence or absence of a minimal zone of the keratinized tissue around dental implants has been a matter of controversy. Many investigators have concluded that attached peri-implant soft tissue does not provide any long-term advantage over alveolar mucosa. However, a growing number of researchers and newer systematic reviews extol its virtue, correlating it with improved soft tissue health, greater patient satisfaction, and fewer complications [1].

Implants are more susceptible to the development of inflammation and subsequent bone loss in the presence of plaque accumulation and bacterial infiltration due to several factors [2]. Narrow zones of keratinized gingiva are less resistant to insult along the implant-mucosa interface. In the presence of an inflammatory response, implants placed in areas with narrow zones of keratinized gingiva have an increased susceptibility to tissue breakdown and showed earlier loss of attachment [3, 4].

In a systematic review that was conducted by Gobbato et al., it was observed that limited keratinized mucosa around implants is associated with clinical parameters of inflammation [5]. However, the predictive value of the keratinized mucosa is limited. There is a need for adequately powered prospective longitudinal clinical trials to prove the importance of the keratinized mucosa in the maintenance of peri-implant health.

In addition to the controversy regarding augmenting the keratinized tissue, Small and Tarnow claimed that recession occurs in 80% of the sites in the first three months after insertion of the dental implant [6]. Furthermore, crestal bone stability is affected by the initial overlying soft tissue thickness as observed by Linkevicius and Apse who noticed that crestal bone resorption up to 1.45 mm may occur if the overlying soft tissue thickness was 2.00 mm or less [7].

Many authors emphasize the importance of the keratinized mucosa to achieve an accepted success rate, and many researchers report that a minimum of 2 mm of keratinized gingiva is needed to achieve healthy gingiva around dental implants [3, 8]. Keratinized gingiva around dental implants is necessary to achieve esthetics, and also it is more resistant to abrasion, recession, less in plaque accumulation and inflammation, and easy to manipulate during stage two surgery and impression making in the prosthetic stage [9]. Furthermore, in the absence of sufficient keratinized mucosa, the pain and difficulty during oral hygiene measurements for the prosthesis could lead to plaque accumulation around the peri-implant tissues and cause discomfort to the patient and mucosal inflammation. The authors have developed many techniques to enhance the soft tissue profile and increase the amount of keratinized gingiva around dental implants; such techniques are apically and laterally repositioned flaps, free gingival grafts, acellular dermal matrix allograft, coronally repositioned flap, and subepithelial connective tissue flaps [10]. The apically repositioned flap has been shown to predictably increase the width of keratinized tissue around natural teeth. The increase in height of the attached gingiva occurs because of an apical alteration of the mucogingival junction which includes apical displacement of the muscular insertions [10]. Although it is well documented that this procedure increases the width of keratinized tissue by 3.5 mm and results in minimal postoperative bone loss, it still has the disadvantage of being unsuitable for a thin biotype gingival tissue, and also the use of sutures to close the flap would produce tension and displace the flap coronally around the implant healing abutment making a problem in controlling the desired positioning of the gingival margin [10]. Regarding the free gingival graft and subepithelial connective tissue graft, respectively, they are applied to cases where there is a shallow oral vestibule. However, these methods have the disadvantage of having a second surgical site and not being an easy procedure to perform by general practitioners [11, 12].

To overcome the limitations of previous surgical techniques, many investigators have devised methods for moving the keratinized gingival flap from the lingual to the buccal and apical directions by fixing a readymade plastic stent to the healing abutment [13, 14].

The present study was performed with the primary objective of clinically assessing and comparing the width of peri-implant keratinized mucosa following the use of a readymade plastic stent with apically repositioned flap versus conventional apically repositioned flap with papillary sparing incisions during single-stage implant placement protocol.

## 2. Materials and Methods

**Figure 1:**
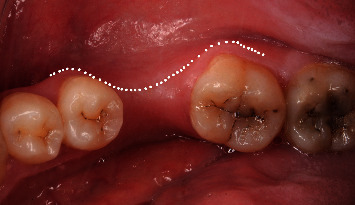
Preoperative occlusal view showing insufficient attached zone around the edentulous space.

The present study enrolled 20 patients with partially edentulous dentition requiring restoration of missing teeth in the premolar-molar region either of maxilla or mandible who were recruited from the outpatient clinic of Oral Medicine, Periodontology, Oral Diagnosis, and Radiology Department, Faculty of Dentistry, Ain Shams University. The purpose of the study was explained to all patients, and informed consent was signed before the conduction of the study. The Faculty of Dentistry Research Ethics Committee had reviewed and accepted the proposal in December 2016 in line with the Helsinki Declaration of 1975. Patients who met the eligibility criteria were randomly allocated using computer-assisted randomization through numbered sealed envelopes into two treatment modality groups: 10 patients were included in Group I (readymade plastic stent) and 10 patients were included in Group II (control). After implant placement and securing of the apically repositioned flap by the readymade plastic stent and sutures in the stent group and control group, respectively, postoperative evaluation at 3-month and 6-month intervals of all clinical parameters (probing depth, plaque index, and bleeding index) in addition to the pink esthetic score was carried out. Patients were selected according to certain inclusion criteria (healthy adult patients, age ranged from 20 to 50 years, and attached gingiva width below 3 mm) (Figure 1), and on the other hand, smokers (>10 cigs/day), pregnant females, and patients with poor oral hygiene or not willing to perform oral hygiene measures were excluded from the study.

### 2.1. Stent Design

The stent is made from polypropylene. The buccal and lingual wings pressing down the flap are 2 mm and 1 mm in size, respectively. The insertion part of the stent had a cylindrical form (diameter: 4.8 mm and height: 2 mm) that could hold the healing abutment by friction between the stent and healing abutment.

### 2.2. Surgical Procedures

Field block of articaine HCl 4% (Septodont Ltd., Septanest 1:100000) containing epinephrine at a concentration of 1 : 100,000 was given buccally and palatally in maxillary surgical sites, while in mandibular surgical sites, nerve block with buccal infiltration anesthesia was given. The surgical approach consisted of a paracrestal papillary sparing incision down to the bone to utilize the keratinized mucosa from the lingual/palatal side. After the lingual incision, leaving 4 mm of keratinized tissue at the buccal flap, a partial-thickness flap was reflected, and an additional vertical incision was made to maximize the apical displacement of the existing keratinized mucosa. After complete reflection of the combined full/partial-thickness flap, a dental implant (Jdental Care, Italy) with suitable diameter and height according to the site was placed in the right position. Healing abutment (Jdental Care, Italy) was placed over the implant and secured with a screwdriver to its final position. For the stent group, the apically repositioned flap was secured around the healing abutment with the help of the readymade plastic stent.

**Figure 2:**
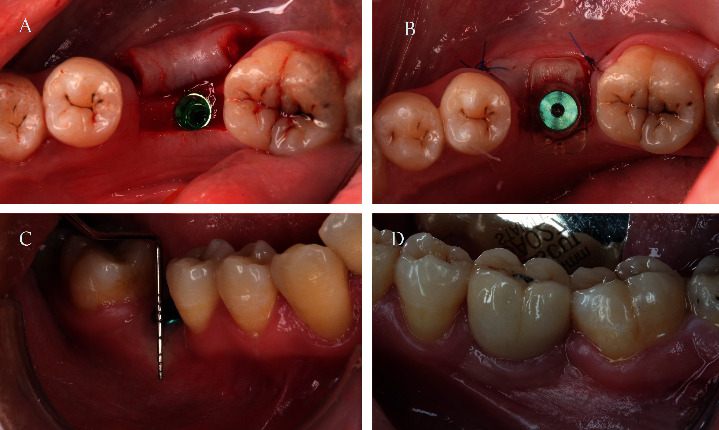
(a) Flap reflection and dental implant placement, (b) healing abutment in place and the flap was secured with the stent, (c) width of KM at 3-month follow-up, and (d) buccal view showing crown in place at 6-month follow-up.

The readymade plastic stent (Louis Button II, Dentis Co., Korea) of suitable diameter to the corresponding healing abutment was used to fit snugly over the healing abutment, and a vertical force was applied to its final position to hold the buccal flap in position and remove the dead space. Originally, the manufacturer recommended that no suture is required and that the displaced flap is mainly stabilized by the stent; however, simple interrupted sutures were made for securing stabilization. For the control group, the apically repositioned flap was secured around the healing abutment with the aid of either simple interrupted sutures over the vertical incisions mesially and distally, or a combination between simple interrupted sutures (Vicryl® 5.0, Polyglactin 910, Ethicon, Johnson & Johnson, Edinburgh, UK) and periosteal sutures for maximum stabilization of the flap. Postoperatively, all patients had received antibiotics for 1 week (amoxicillin + clavulanic acid 1000 mg b.d.s) (Amoxil MUP Egypt) and an anti-inflammatory drug (ibuprofen b.d.s) (Amoun, Egypt). Patients were instructed to pass the first 24 hours and to start rinsing twice daily with a 0.12% chlorhexidine digluconate (Antiseptol Kahira Pharm, Egypt) mouth rinse and to avoid mechanical plaque control at the site of surgery for 15 days. Patients were instructed not to brush the surgical area for 2 weeks (Figure 2).

For the stent group, the readymade plastic stent was removed after 10 days according to manufacturer instructions [14]. For the control group, sutures were removed after 2 weeks.

After 15 days, patients were instructed to use the Bass technique for tooth brushing.

### 2.3. Statistical Analysis

The mean and standard deviation values were calculated for each group in each test. Data were explored for normality using Kolmogorov–Smirnov and Shapiro–Wilk tests; probing depth, bleeding index, plaque index, width of attached gingiva, and pink esthetic score showed nonparametric (not normal) distribution while bone width and bone height showed parametric (normal) distribution. For nonparametric data, Mann–Whitney was used for comparison between two groups in nonrelated samples. Friedman was used for comparison between more than two groups in related samples. Wilcoxon was used for comparison between two groups in related samples. For parametric data, independent sample *t*-test was used for comparison between two groups in nonrelated samples. Repeated-measures ANOVA was used for comparison between more than two groups in related samples. Paired sample *t*-test was used for comparison between two groups in related samples. The significance level was set at *p* ≤ 0.05. Statistical analysis was performed with IBM® SPSS® Statistics version 20 for Windows.

## 3. Results

**Figure 3:**
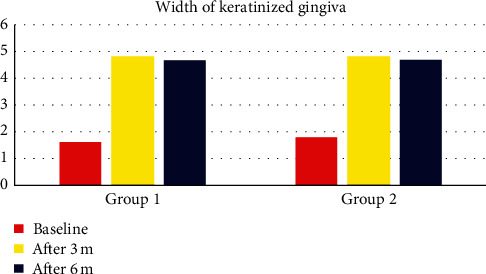
Bar chart representing the width of keratinized gingiva for different groups at different time intervals.

The results of the present study revealed that the values of PD, PI, and BI were not statistically significantly different between the stent group and the control group at baseline and 3-month and 6-month follow-up period. Furthermore, no significant change was evident between each time interval within each group, indicating that the preimplant mucosa was maintained in a healthy condition over the whole study period. This study also demonstrated a statistically significant gain in the width of KM between baseline and each of 3-month and 6-month intervals in the stent group, but no statistically significant difference was found between 3-month and 6-month intervals. The mean width of KM was increased from 1.60 mm at baseline to the value of 4.85 mm at 3 months' interval and 4.70 mm at 6 months' interval. Also, in the control group, there was a statistically significant gain in the width of KM between baseline and each of 3-month and 6-month intervals. The mean width of KM was increased from 1.80 mm at baseline to the value of 4.85 mm at 3 months' interval and 4.70 mm at 6 months' interval (Figure 3).

No statistically significant difference was found between the stent group and control group in 6 months' interval where *p*=0.840, where both groups showed the same mean value (4.70 ± 0.35 and 4.70 ± 0.63, respectively). The percent of change in the width of KM was found to be higher in the stent group than in the control group with no statistical significance. There was a statistically significant difference in the pink esthetic score between baseline, 3-month, and 6-month intervals in each group (*p* < 0.001). However, there was no statistically significant difference between the two groups in the same time intervals. The highest mean value in group 1 was found in 6 months' interval (7.90 ± 1.10) followed by 3 months' interval (4.90 ± 0.74), while the least mean value was found in baseline interval (0.00 ± 0.00). In group 2, the highest mean value was found in 6 months' interval (8.00 ± 0.0.94) followed by 3 months' interval (4.80 ± 0.42), while the least mean value was found in baseline interval (0.00 ± 0.00) (Table 1).

## 4. Discussion

The absence of keratinized gingiva around teeth and the resulting mobility of marginal tissues promote bacterial invasion of the gingival sulcus. Peri-implant tissues lack the perpendicular arrangement of the supracrestal collagen fibers, thereby creating a much weaker mechanical attachment compared to natural teeth. Hence, a wider keratinized tissue zone ensures the long-term success of the dental implant [1, 15].

In the present study, single-stage implant placement was applied to take the advantage of one-stage surgical procedure, less chair time, less pain, and shorter healing period. This was supported by Buser and Lang in their study as they concluded that the nonsubmerged placement of dental implants offers several advantages from biologic, clinical, and biomechanical points of view; therefore, this approach has been increasingly utilized by clinicians in recent years [16].

During the implant placement phase, APF was performed to increase the width of KM using the technique described by Friedman [17], in addition to two modifications seeking better healing outcomes. The first modification was using a combined full/partial-thickness flap instead of a full-thickness flap only. This was based on the study that reported that the partial-thickness flap has a clinically relevant advantage over the full-thickness flap. They reported that partial-thickness flap results in less bone loss than do full-thickness flap [18]. The second modification in our surgical technique was to use a papillary sparing paracrestal incision instead of the conventional sulcular incision as papillary sparing incision seems to trigger less postoperative gingival reduction than sulcular one [18]. Furthermore, it also protects the marginal gingiva of the adjacent teeth to prevent the subsequent crestal bone loss as clearly illustrated by Binderman et al. who reported significant bone loss initiated on the periodontal ligament aspect of the alveolar bone when the marginal gingiva was incised in a coronal approach [19]. Moreover, Greenstein and Tarnow concluded that papillary sparing incisions facilitated accessing the bone to place implants and restore compromised osseous and gingival architecture. Their major benefit was to enable surgical procedures to be performed without inducing recession of papillae adjacent to treated sites [20].

Modifications performed in the apically repositioned flap in the current study were not identically performed before in other publications, so an exact comparison with other studies is not possible. The results of the present study revealed that the values of PD, PI, and BI were not statistically significantly different between the stent group and control group at baseline and 3-month and 6-month follow-up period. Furthermore, no significant change was evident between each time interval within each group, indicating that the preimplant mucosa was maintained in a healthy condition over the whole study period. This study also demonstrated a statistically significant gain in the width of KM between baseline and each of 3-month and 6-month intervals in the stent group, but no statistically significant difference was found between 3-month and 6-month intervals. The mean width of KM was increased from 1.60 mm at baseline to the value of 4.85 mm at 3 months' interval and 4.70 mm at 6 months' interval. Also, in the control group, there was a statistically significant gain in the width of KM between baseline and each of 3-month and 6-month intervals. The mean width of KM was increased from 1.80 mm at baseline to the value of 4.85 mm at 3 months' interval and 4.70 mm at 6 months' interval. The percent of change in the width of KM was found to be higher in the stent group than in the control group with no statistical significance.

These results are in accordance with other studies that reported a gain of KM around implants. One study reported that the technique of APF around implants showed a statistically significant improvement with a mean gain of 3.95 mm in the width of KM at the end of 12 weeks [10]. Similarly, a case report showed a gain in KG following APF over the whole study period [14]. Another study reported that peri-implant keratinized mucosa demonstrated a clinical gain in all cases over the entire follow-up period [21]. Also, in line with our study, another investigation evaluated the effects of the use of a readymade plastic stent on the width of peri-implant keratinized mucosa and soft tissue and reported that the width of the keratinized mucosa was significantly higher and the distance from the top of the implant platform to the mucogingival junction was significantly longer in the readymade plastic stent group [12]. On the other hand, Kim et al. reported a decrease in the width of keratinized mucosa 1 and 3 months after surgery in both control and test groups with a greater amount of reduction in the control group [13].

To the best of our knowledge, this is the first study to assess the esthetic outcome of apically displaced flap around implants using PES. The assessment of esthetic outcome using PES gave an average score of 7.9 after 6 months, indicating good esthetics, and this value was statistically higher in both groups than each of the 3-month and baseline values. On the other hand, no statistically significant difference was found between both groups in 6 months which was in accordance with a study which evaluated the esthetic outcome of early placed maxillary anterior single tooth implant and reported a mean total PES of 7.8 [22]. This is not surprising because the PES is mainly influenced by the local anatomy and the applied surgical procedure to regenerate the peri-implant soft tissue.

APF with a partial-thickness flap is preferred when a minimum band of existing keratinized mucosa exists. This technique requires no graft or complicated sutures and causes less pain to the patient. The method also reduces the overall operation time and produces acceptable results. However, the periosteal sutures used in this method to stabilize the displaced flap are technique-sensitive and time-consuming and lack vertical pressure over the displaced flap, creating dead space. This context delays the healing process and sometimes induces necrosis. Therefore, the prefabricated implant-retained stent was designed to overcome these shortcomings and to be used easily in general clinics without any laboratory equipment [14]. Within the limitation of this study, the readymade stent had the following benefits: the stent secured the displaced labial flap buccoapically with the existing keratinized mucous band which resulted in flattening the flap over the underlying periosteum and prevented shrinkage of the gained new keratinized tissue; the stent reduced the operation time and effort critically due to no need for sutures; moreover, the secondary healing area was partially covered, so food impaction or pain was decreased.

## 5. Conclusions

The use of a readymade plastic stent in combined full/partial-thickness apically repositioned flap shows to be effective in increasing the width of KM compared to the conventional technique with substantially reduced time and effort.

## Figures and Tables

**Table 1:** The mean and standard deviation (SD) values of pink esthetic score of different groups.

Variables	Pink esthetic score						
	Group1			Group2			*p* value
	Mean	SD	Median	Mean	SD	Median	
Baseline	0.00^cA^	0.00	0.00	0.00^cA^	0.00	0.00	**1** ^**ns**^
After 3 m	4.90^bA^	0.74	5.00	4.80^bA^	0.42	5.00	**0.788** ^**ns**^
After 6 m	7.90^aA^	1.10	8.00	8.00^aA^	0.94	8.00	**0.935** ^**ns**^
*p* value	**<0.001** ^*∗*^			**<0.001** ^*∗*^			

Means with different small letters in the same column indicate a statistically significant difference. Means with different capital letters in the same row indicate a statistically significant difference. ^*∗*^Significant (*p* < 0.05); ns: nonsignificant (*p* > 0.05).

## Data Availability

The data are available upon request from the corresponding author.
